# A Piezoelectric Ionic Cocrystal of Glycine and Sulfamic Acid

**DOI:** 10.1021/acs.cgd.1c00702

**Published:** 2021-09-27

**Authors:** Sarah Guerin, Sanaz Khorasani, Matthew Gleeson, Joseph O’Donnell, Rana Sanii, Reabetswe Zwane, Anthony M. Reilly, Christophe Silien, Syed A.M. Tofail, Ning Liu, Michael Zaworotko, Damien Thompson

**Affiliations:** † SSPC, Science Foundation Ireland Research Centre for Pharmaceuticals, University of Limerick, V94 T9PX Limerick, Ireland; ‡ Department of Physics, Bernal Institute, University of Limerick, V94 T9PX Limerick, Ireland; § Department of Chemical Sciences, Bernal Institute, University of Limerick, V94 T9PX, Limerick, Ireland; ∥ School of Chemical Sciences, Dublin City University, Glasnevin, D09 C7F8 Dublin, Ireland

## Abstract

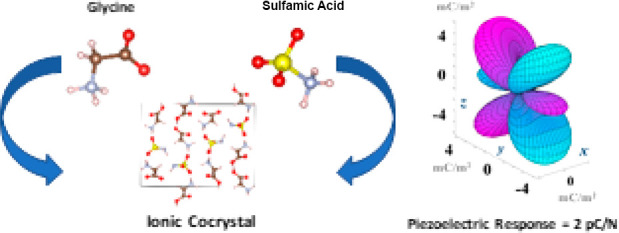

Cocrystallization of two or more molecular compounds can dramatically change the physicochemical properties of a functional molecule without the need for chemical modification. For example, coformers can enhance the mechanical stability, processability, and solubility of pharmaceutical compounds to enable better medicines. Here, we demonstrate that amino acid cocrystals can enhance functional electromechanical properties in simple, sustainable materials as exemplified by glycine and sulfamic acid. These coformers crystallize independently in centrosymmetric space groups when they are grown as single-component crystals but form a noncentrosymmetric, electromechanically active ionic cocrystal when they are crystallized together. The piezoelectricity of the cocrystal is characterized using techniques tailored to overcome the challenges associated with measuring the electromechanical properties of soft (organic) crystals. The piezoelectric tensor of the cocrystal is mapped using density functional theory (DFT) computer models, and the predicted single-crystal longitudinal response of 2 pC/N is verified using second-harmonic generation (SHG) and piezoresponse force microscopy (PFM). The experimental measurements are facilitated by polycrystalline film growth that allows for macroscopic and nanoscale quantification of the longitudinal out-of-plane response, which is in the range exploited in piezoelectric technologies made from quartz, aluminum nitride, and zinc oxide. The large-area polycrystalline film retains a damped response of ≥0.2 pC/N, indicating the potential for application of such inexpensive and eco-friendly amino acid–based cocrystal coatings in, for example, autonomous ambient-powered devices in edge computing.

## Abstract 2

Glycine and sulfamic acid form an ionic cocrystal that demonstrates a measurable longitudinal piezoelectric response in single-crystal and polycrystalline form. The crystals are characterized using piezoresponse force microscopy and second harmonic generation, and the material properties are rationalized and quantified using density functional theory calculations.

## Introduction

Bioderived and bioinspired structural materials have emerged as high-performance piezoelectrics that generate charge under an applied force due to their noncentrosymmetric crystal structures.^1,2^ However, the predominance of shear piezoelectricity in organic crystals (and polymers) hinders their deployment in commercial sensors, as very few crystallize in a space group that allows for a longitudinal out-of-plane response,^3^ typically *d*_33_, that is readily exploited in standard device architectures. Crystal engineering of amino acid based crystalline materials may provide a solution, as the three glycine polymorphs have served as a model for using simple chemical modifications to create and improve electromechanical properties.^4^ Glycine, the only nonchiral essential amino acid, crystallizes in the nonpiezoelectric centrosymmetric α polymorph from aqueous solution,^5^ but the addition of a salt to the crystallization bath induces the noncentrosymmetric γ-polymorph with a maximum response of 10 pC/N,^4,6,7^ and crystallization in alcohol affords a metastable β-polymorph with a massive shear piezoelectric response of 180 pC/N.^4^ A centrosymmetric δ-polymorph of glycine has also been reported at high pressures. The noncentrosymmetric glycine polymorphs, as well as amino acids, more broadly also demonstrate unique and extremely useful optical and nonlinear optical properties.^8−12^ Both β-glycine and γ-glycine have been combined with biocompatible polymers for piezoelectric actuation^13^ and wound healing.^14^

Cocrystallization has been used to change the composition of solid forms of organic molecules through the formation of multicomponent crystal forms involving the target functional molecule and a second molecule, a coformer, with complementary functional groups.^15,16^ This approach has been used to create drug substances with superior properties, including higher melting point, improved tabletability, solubility, stability, bioavailability, and permeability. Importantly, molecular properties such as efficacy against a biological target can be preserved or enhanced, a key issue if the API shows poor aqueous solubility and low oral bioavailability, with several cocrystal drug products being introduced to the market in recent years.^17,18^ While the first piezoelectric cocrystals were reported in 1946,^19^ it is only in the past decade that cocrystallization has been exploited to alter the electromechanical and optical properties of organic crystals,^20,21^ endowing them with properties such as exceptionally high elastic moduli^22^ and a large piezoelectric response.^23,24^ Glycine cocrystallized with 2D materials (BN, MoS_2_, and WS_2_) has also demonstrated piezoelectricity.^25^

Here, we show that two molecules that crystallize independently in centrosymmetric space groups from an undoped aqueous solution can form a noncentrosymmetric cocrystal due to interactions that generate acentric hydrogen-bonded motifs. The existence of such motifs or “supramolecular synthons” is key to understanding cocrystal formation and provides design of new cocrystals from first principles.^16^ In previous studies,^26,27^ both the functional molecule and the coformer each crystallized in noncentrosymmetric space groups so that, whereas cocrystallization modulatedthe existing properties, it did not create a new functionality. Notably, herein the space group change from centrosymmetric to noncentrosymmetric was made solely via the cocrystallization of achiral coformers. We also highlight the use of polycrystalline film growth to increase the size and quality of crystals for electromechanical measurements and exploitation in piezoelectric device applications, and the use of second-harmonic generation (SHG) measurements as a suitable noncontact characterization technique. SHG microscopy is an effective tool for studying chiral crystals and has been utilized to study pharmaceutical compounds,^28^ including glycine single crystals^29^ and cocrystals.^30^ Our experimental SHG measurements are guided and substantiated by DFT models and further validated by piezoresponse force microscopy (PFM) and show that cocrystallization alone is sufficient to create new functional properties in organic crystals. Through the specific example of engineering a significant longitudinal piezoelectricity of ∼2 pC/N in α-glycine cocrystals, we demonstrate a simple, inexpensive, and widely applicable means of expanding the range of technology applications of biopiezoelectric materials.

## Materials and Methods

### Crystal Growth

Glycine and sulfamic acid powders were dissolved in a 2:1 molar ratio in aqueous solution. The solution was allowed to evaporate slowly at 50 °C, and single crystals suitable for single-crystal X-ray diffraction formed within 3–4 days. For polycrystalline film growth, the glycine–sulfamic acid solution was stirred thoroughly and deposited on clean copper and brass substrates using a pipet to form a liquid film on the surface. Samples were left to evaporate for up to 24 h under ambient conditions until dense solid films had formed.

### Single-Crystal X-ray Diffraction

The crystal structure of the glycine–sulfamic acid 2:1 ionic cocrystal was determined at −100 °C on a Bruker D8 Quest fixed-χ single-crystal diffractometer equipped with a sealed-tube X-ray source that delivers Mo Kα (λ = 0.71073 Å), a TRIUMPH monochromator, a PHOTON 100 detector, and a nitrogen-flow Oxford Cryosystem attachment. Unit cell determination, data reduction, and absorption correction (multiscan method) were conducted using the Bruker APEX3 suite.^31^ Using Olex2,^32^ the structure was solved with the ShelXT structure solution program^33^ using intrinsic phasing and refined with the ShelXL refinement package^34^ using least-squares minimization.

### Density Functional Theory

All modeling was performed using the Vienna Ab initio Simulation Package (VASP) code^35^ for periodic density functional theory calculations (DFT)^36^ with plane wave basis sets^37^ and the projector augmented-wave (PAW) method.^38^ Exchange-correlation effects were treated using the Perdew, Burke, and Ernzerhof (PBE)^39^ implementation of the generalized gradient approximation (GGA).^40^ All crystal structures were optimized using conjugate gradient minimization^41^ with a 6 × 4 × 2 γ-centered *k*-point grid and a plane-wave energy cutoff of 600 eV. The stiffness tensor was calculated using a finite differences method, with each atom displaced in each direction by ±0.01 Å, with 2 × 2 × 2 *k*-point sampling and a plane-wave energy cutoff of 800 eV. All calculations were carried out using the tetrahedron smearing method. Piezoelectric strain constants and dielectric tensors were calculated using density functional perturbation theory (DFPT).^42^ The ratio of the piezoelectric charge coefficients, *e*, which are calculated directly by VASP, and the elastic stiffness constants, *c*, give the piezoelectric strain coefficients, *d*. These constants are indexed as *d*_*ij*_, where *i* is the direction of the applied stimulus and *j* the direction of the corresponding electromechanical response. Young’s moduli are presented as Voigt–Reuss–Hill averages,^43^ calculated using the ELATE application.^44^

### Longitudinal Piezoelectric Measurements

Longitudinal piezoelectric constants were measured using a commercial PiezoTest *d*_33_ meter, with an accuracy of 0.01 pC/N (pm/V).

### Optical Microscopy

Optical images were taken using an Olympus BX51 light microscope connected to an Olympus SC50 digital camera.

### Piezoresponse Force Microscopy

For the PFM characterization of biomolecular crystals, an NT-MDT Ntegra Spectra scanning probe microscope was used with platinum-coated probes with a high spring constant of 19.57 N/m to remove electrostatic interactions.^45^ The microscope was operated in contact mode, with an AC voltage applied between the conductive probe and grounded sample at a frequency of 21 kHz. This frequency is well below the contact resonance of the tip–sample system, avoiding any artificial amplification of the signal.

### Second-Harmonic Generation

#### Setup

Second-harmonic generation (SHG) was measured in transmission mode using a Spark Antares fiber laser (1064 nm, 80 MHz, 5 ps) with a power of 75 mW incident on the sample. The numerical apertures (NA) of the excitation and collection objectives were 0.3NA and 0.4NA, respectively. The substrate was raster-scanned over a 100 μm × 100 μm area using a PI E-664 scanner on a PI Nanocube XYZ piezo stage. The excitation was linearly polarized in the horizontal direction. SHG and linear scattering (LS) were simultaneously recorded by a pair of Hamamatsu H11901-20 photomultiplier tubes (PMT) connected to Hamamatsu C7319 preamplifiers, with Semrock FF01-535/150–25 and Thorlabs FESH600 filters placed in front of the PMT measuring SHG to filter out the excitation beam. A schematic of the microscope is shown in Figure 3a.

#### Effective Nonlinear Susceptibility Quantification

To verify that the signal recorded by the PMT is SHG, the SHG intensity was measured as a function of excitation power. As SHG is a second-order nonlinear process, the signal intensity scales according to *P*^2ω^ ∝ *aP*_*i*_^*b*^, where *P*^2ω^ is the recorded SHG signal at frequency 2ω and *P*_*i*_ is the excitation power with *a* and *b* numerical constants, where the value of *b* should be close to 2. The LS images were thresholded to separate the objects from the background to create a mask. Applying this mask to the SHG image, we integrated the pixel intensity within each object to find a value for SHG. Assuming the particles are roughly spherical in shape, we can approximate the radius of each particle using *A* = *πr*^2^ by assuming an “equivalent sphere” scattering cross section.^46^ SHG is then quantified in units of pm/V by comparison to KDP (potassium dihydrogen phosphate, KH_2_PO_4_) particles of the same size, measured under the same excitation conditions.^47,48^ Possible phase-matched particles are excluded on the basis of having SHG per square micrometer greater than 3 times the standard deviation of SHG in the reference data set,^49^ and only particles with effective radii within 2% of each other are compared. Given the pixel size of ∼0.4 μm per pixel, this provides a comparison of particles with effectively the same area and hence the same radius. Given the large depth of field (12.5 μm) for our setup (20×, 0.3 NA, 1064 nm) relative to the sub 5 μm radius of the particles tested, we can assume that the full volume of particle scanned per pixel can contribute to the measured signal.^49^ The effective susceptibility *d*_eff_ is calculated using the equation
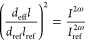
1where *l* and *l*_ref_ are the lengths of the sample and reference material, respectively (we take the length to be proportional to the radius of the particles), *d*_ref_ is the effective susceptibility of KDP, and *I*^2ω^ and *I*_ref_^2ω^ are SHGs measured from the sample and reference materials, respectively. This analysis allows a value of *d*_eff_ to be determined for each identified particle through a comparison to the reference material KDP with a known SHG susceptibility of 0.38 pm/V.^50^

## Results and Discussion

Sulfamic acid crystallizes with *D*_2*h*_ point group symmetry in space group No. 61, *Pbca*,^51,52^ while α-glycine crystallizes in monoclinic space group No. 14, *P*2_1_/*c*, both of which are centrosymmetric and so preclude a nonzero piezoelectric response. Cocrystallization results in a noncentrosymmetric glycine–sulfamic acid salt (monoclinic space group no. 7, *P*1*c*1) that provides 10 nonzero piezoelectric strain constants, *d*_*ij*_. As the electromechanical response depends on both polarization and elastic stiffness, DFT calculations were carried out to predict the full piezoelectric and elastic tensors of the crystals. Dielectric constants ε were also calculated and combined with the piezoelectric tensor to predict the obtainable output voltage constants, *g*_*ij*_ = *d*_*ij*_/ε.

**Figure 1:**
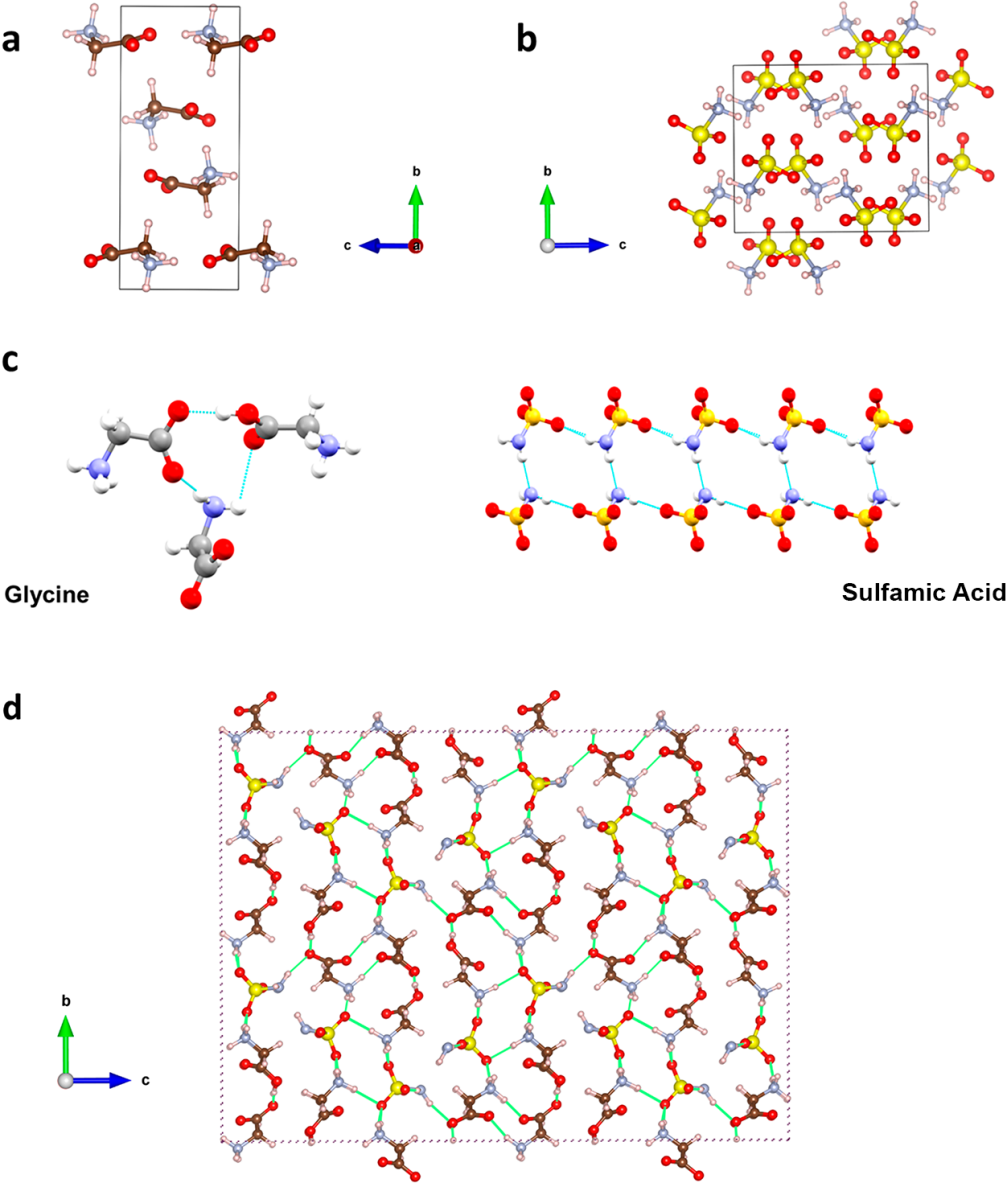
Noncentrosymmetric crystal structure of the 2:1 glycine–sulfamic acid ionic cocrystal. The centrosymmetric unit cells of (a) α-glycine and (b) sulfamic acid and (c) the noncentrosymmetric hydrogen-bonded motifs formed by adjacent [(Gly)_2_H]^+^ cations (left) and adjacent H_2_NSO_3_^–^ anions (right). (d) Cocrystal packing directed by a hydrogen bond network (2 × 2 × 2 supercell with hydrogen bonds shown in green). Sulfur atoms are shown in yellow, hydrogens in white, oxygens in red, carbons in brown/gray, and nitrogens in blue/lilac.

The experimentally determined cocrystal structure reveals that it is a 2:1 glycine to sulfamic acid cocrystal in which one glycine molecule has been protonated by sulfamic acid to afford an anhydrous ionic cocrystal of the composition [(Gly)_2_H]^+^[NH_2_SO_3_]^−^ (Figure 1). An analysis of the crystal packing, in particular, the self-assembly of both [(Gly)_2_H]^+^ cations and sulfamate anions, helps to explain why [(Gly)_2_H]^+^[NH_2_SO_3_]^−^ adopts a noncentrosymmetric crystal-packing pattern. Specifically, as revealed by Figure 1c, [(Gly)_2_H]^+^ cations form a three-component charge-assisted hydrogen-bonded motif involving the ammonium group of an adjacent [(Gly)_2_H]^+^ cation, whereas sulfamate anions form hydrogen-bonded tapes sustained by NH···O hydrogen bonds. In both cases, a center of inversion is precluded by the observed motifs.

The glycine–sulfamic acid cocrystal is predicted to have low elastic anisotropy (Table 1), with longitudinal elastic stiffness constants of 23–33 GPa and shear constants of 5–7 GPa. The predicted Young’s modulus of 17 GPa is comparable to that of the β-glycine polymorph predicted using the same methods.^4^ The predicted piezoelectric charge constants are much lower than those found in β-glycine or γ-glycine, which showed maximum *e*_*ij*_ values of 0.26 and 0.83 C/m^2^, respectively. The maximum cocrystal value is a transverse constant of −0.10 C/m^2^, which results in a piezoelectric strain constant *d*_12_ of −3.7 pC/N (Table 2). Most importantly, the cocrystal shows a predicted longitudinal *d*_33_ constant of −2.0 pC/N, which is novel among amino acid crystals and their derivatives. Only the trigonal γ-glycine polymorph crystallizes in a space group that permits a nonzero *d*_33_ response, which is 10 pC/N in single-crystal form^6,7^ and 1 pC/N in polycrystalline form.^53^

**Table 1:** Predicted Dielectric and Elastic Properties of the Glycine–Sulfamic Acid Cocrystal and Its Crystallized Constituents

elastic constant (GPa)	cocrystal	sulfamic acid	glycine
*c*_11_	32.5	34.5	53.7
*c*_22_	28.0	52.4	21.7
*c*_33_	22.7	42.9	71.5
*c*_44_	5.8	14.3	7.5
*c*_55_	4.8	12.8	16.3
*c*_66_	7.1	18.1	6.0
Young’s modulus (exptl)	17 (N/A)	34 (25–35)	30 (30–36)

dielectric constant	cocrystal	sulfamic acid	glycine
ε_1_	2.32	2.52	2.74
ε_2_	2.27	2.56	2.19
ε_3_	2.32	2.47	2.60
ε_r_ (exptl)	2.30	2.52 (3.16)	2.51 (3.21)

**Table 2:** Predicted Piezoelectric Constants of the Glycine–Sulfamic Acid Cocrystal

tensor component	charge constant (C/m^2^)	strain constant (pC/N)	voltage constant (mV m/N)
*d*_11_	–0.061	–1.9	–36
*d*_12_	–0.104	–3.7	–72
*d*_13_	0.048	2.1	41
*d*_31_	0.033	1.0	24
*d*_32_	–0.007	–0.25	–6
*d*_33_	–0.046	–2.0	–49
*d*_15_	0.006	1.3	26
*d*_24_	0.015	2.6	50
*d*_26_	0.001	0.14	2
*d*_34_	–0.022	–4.7	–112

Predicted glycine values are taken from ref (4). Experimental values for Young’s moduli and dielectric constants are given in parentheses and are taken from refs (4 and 54−56)

**Figure 2:**
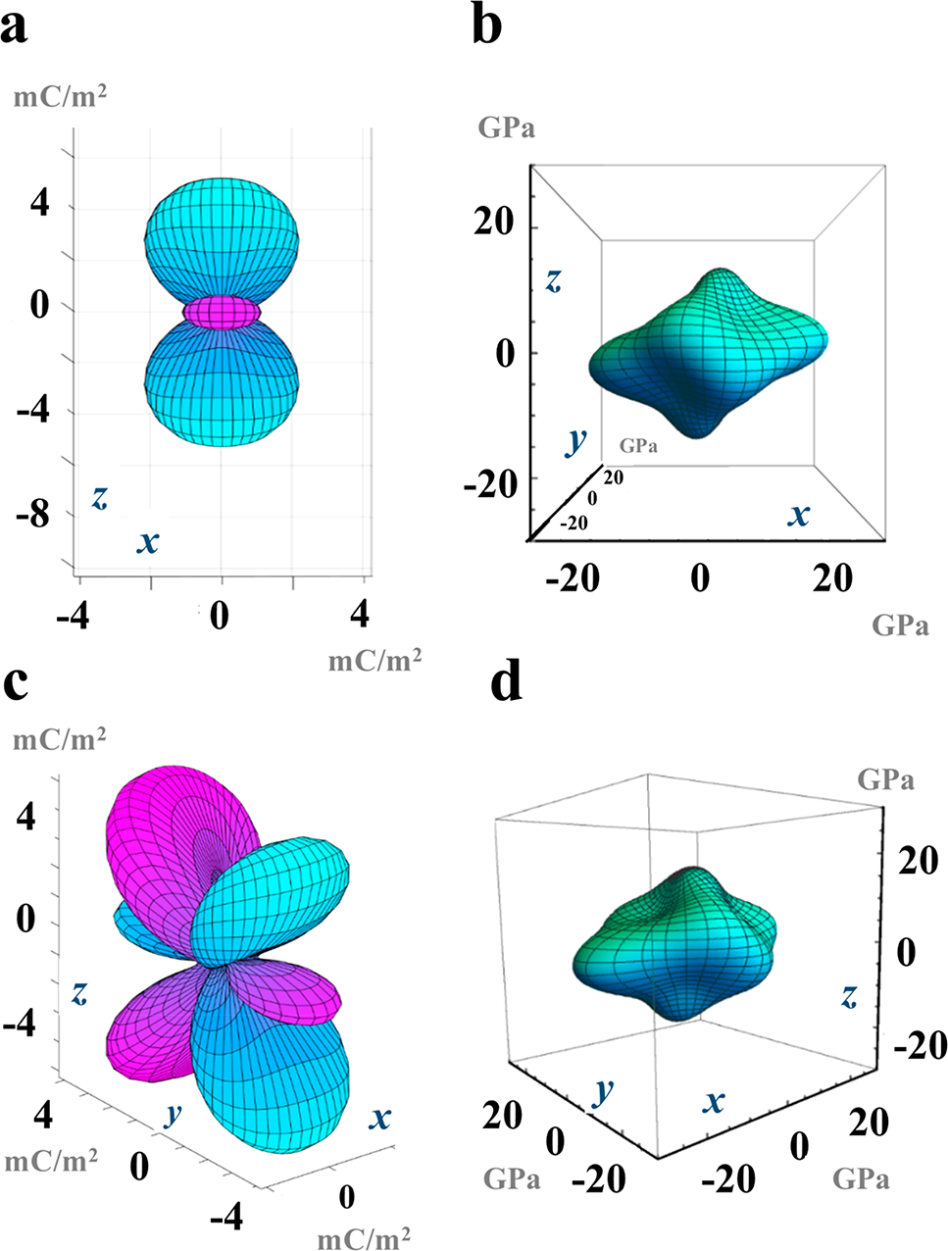
Computed directional dependence of the electromechanical response of glycine–sulfamic acid cocrystals. (a) Piezoelectric polarization and (b) Young’s moduli in the *ac* plane (top panels) and (c, d) oriented at an angle to show the 3D features.

The glycine–sulfamic acid cocrystal assembles via a charge-assisted NH ···O (2.00 Å) and OH ···O (1.59 Å) hydrogen-bond network. The hydrogen bonds are strongly oriented in the *a* and *c* directions (Figure 1), which coincide with the local maxima of the Young modulus (Figure 2). This indicates that the ionic cocrystal shows resistance in the direction of the hydrogen bonds, confirming the directional influence of hydrogen bonds on the mechanical response of the crystal. Figure 2 also presents the directional dependence of the piezoelectric polarization (in units of mC/m^2^) in the glycine–sulfamic acid cocrystal, alongside the Young modulus. The polarization magnitude and thus the piezoelectric response are generally larger in directions where the Young modulus is small. This means that the ionic cocrystal shows piezoelectric response in directions where there is negligible uniaxial resistance. This finding is consistent with earlier claims that hydrogen-bond networks facilitate a change of dipole moment during the deformation of biomolecular crystals,^57,58^ necessary for a significant piezoelectric response, with deformation in the direction of the hydrogen bonds inhibiting the piezoelectric response.

**Figure 3:**
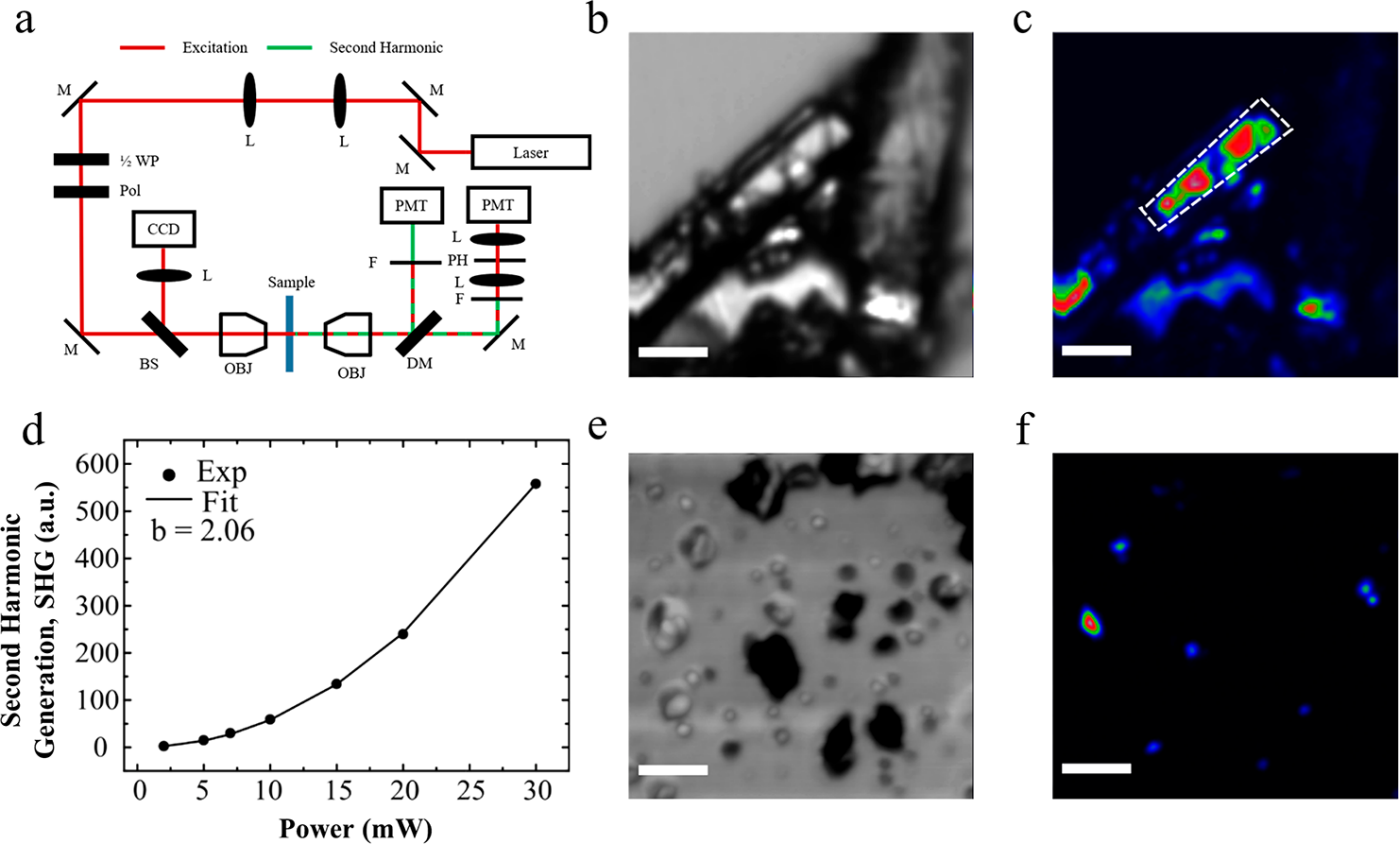
SHG measurements of glycine–sulfamic acid cocrystals. (a) Schematic of the SHG transmission microscope: M, mirror; L, lens’ 1/2 WP, half-wave plate; Pol: polarizer; BS, beam splitter; OBJ, objective lens; DM, dichroic mirror; F, filter; PH, pinhole; PMT, photon multiplier tube. (b) Linear scattering (LS) from cocrystal needle. The scale bar on this and all images is 20 μm. (c) SHG intensity map of the same sample area with the dashed white outline showing the integration area used to make (d). (d) SHG signal vs incident power. (e) LS image of cocrystal particles. (f) SHG intensity map of the same area.

Single crystals were grown via a standard evaporation methodology from aqueous solution (see Materials and Methods) but persistently grew as dense three-dimensional clusters, even after drying, filtering, and recrystallization. While a small number of high-quality crystals could be isolated for structural characterization, neither these nor the crystal clusters were suitable for electromechanical characterization due to their size and fragility: i.e., they could not withstand rgw application of mechanical force in piezoresponse force microscopy (PFM) and piezometer measurements. It should be noted that this is not due to the inherent mechanical properties of the cocrystal but rather is due to the crystal growth mechanisms. A thermal analysis of the cocrystals (Figures S1 and S2) shows that they demonstrate good thermal stability, with no loss of weight until 473 K, corresponding to an exothermic heat release. Two endothermic peaks are also observed at approximately 398 and 523 K. Noncontact second-harmonic generation (SHG) measurements were carried out using a laser transmission microscope (Figure 3a) to investigate their nonlinear optical properties, including piezoelectric response.

An LS image of a fragment of a cocrystal is shown in Figure 3b, with its corresponding SHG response being given in Figure 3c. There is a clear SHG signal generated from the smooth section of the cocrystal. Less SHG is recorded from the rougher sections of the crystal because the excitation is scattered away rather than propagates through the material where it can generate second harmonics: i.e., the darker patches in the LS image. To check that the recorded signal is true SHG, we integrated the pixel values within the dashed white box in Figure 3c and plotted it as a function of increasing excitation power (Figure 3d). Using numerical fitting, we find a value of *b* = 2.06 ± 0.01, which is close to the expected value of 2, confirming that the signal recorded is SHG arising from the noncentrosymmetric structure of the cocrystal.

After confirming that particles of the cocrystal produce SHG, we prepared a bulk sample by mechanically grinding into a fine powder to quantify the effective nonlinear susceptibility. LS and SHG images are shown in Figure 3e,f, respectively. Particles with larger SHG response appear more transparent in the LS images, meaning there is less contrast between the particle and glass substrate. This indicates that the beam is less scattered when propagating through the particle. The sharper contrast (darker) in LS is likely due to low crystallinity and roughness. The random orientation of the particles with respect to the incident laser varies the response. The small particle sizes typical of organic materials are beneficial as the effects of phase matching and coherence lengths are negligible, which allows decoupling between the linear and nonlinear optical effect before quantification. To obtain a reasonable statistical estimate of the effective nonlinear susceptibility, we measured the response of 40 different particles, using the approach detailed in Methods.

The maximum effective nonlinear susceptibility value was 1.8 pm/V (Table 3), relative to the reference material KDP. There is a large variation of susceptibilities found, due to the nonuniformity of the cocrystal microparticles tested. The mean effective longitudinal value from SHG is 0.57 pm/V. This is a larger response in comparison to KDP^50^ (0.38 pm/V) but smaller than that of urea^59^ (1.04 pm/V). Note that SHG is mostly an electronic effect, while the DFT value combines electronic and ionic contributions to the strain tensor. Isolating the electronic contribution to the DFT *d*_33_ value also yields 0.57 pm/V, which indicates that the full material response (*d*_33_ = −2.0 pC/N; Table 2, confirmed by PFM on polycrystalline films) is primarily due to ionic deformation. The precise agreement between the electronic DFT value and the mean SHG value is likely fortuitous, given the large error bar of the measurements, but the general agreement illustrates the predictive power of the DFT model.

**Table 3:** Effective Nonlinear Susceptibility Values (pm/V) Extracted from SHG Measurements (*n* = 40)^a^

minimum	0.07
maximum	1.77
mean	0.57
standard deviation	0.48

a The SHG values in pm/V and DFT values in pC/N (Table 2) have equivalent units.

**Figure 4:**
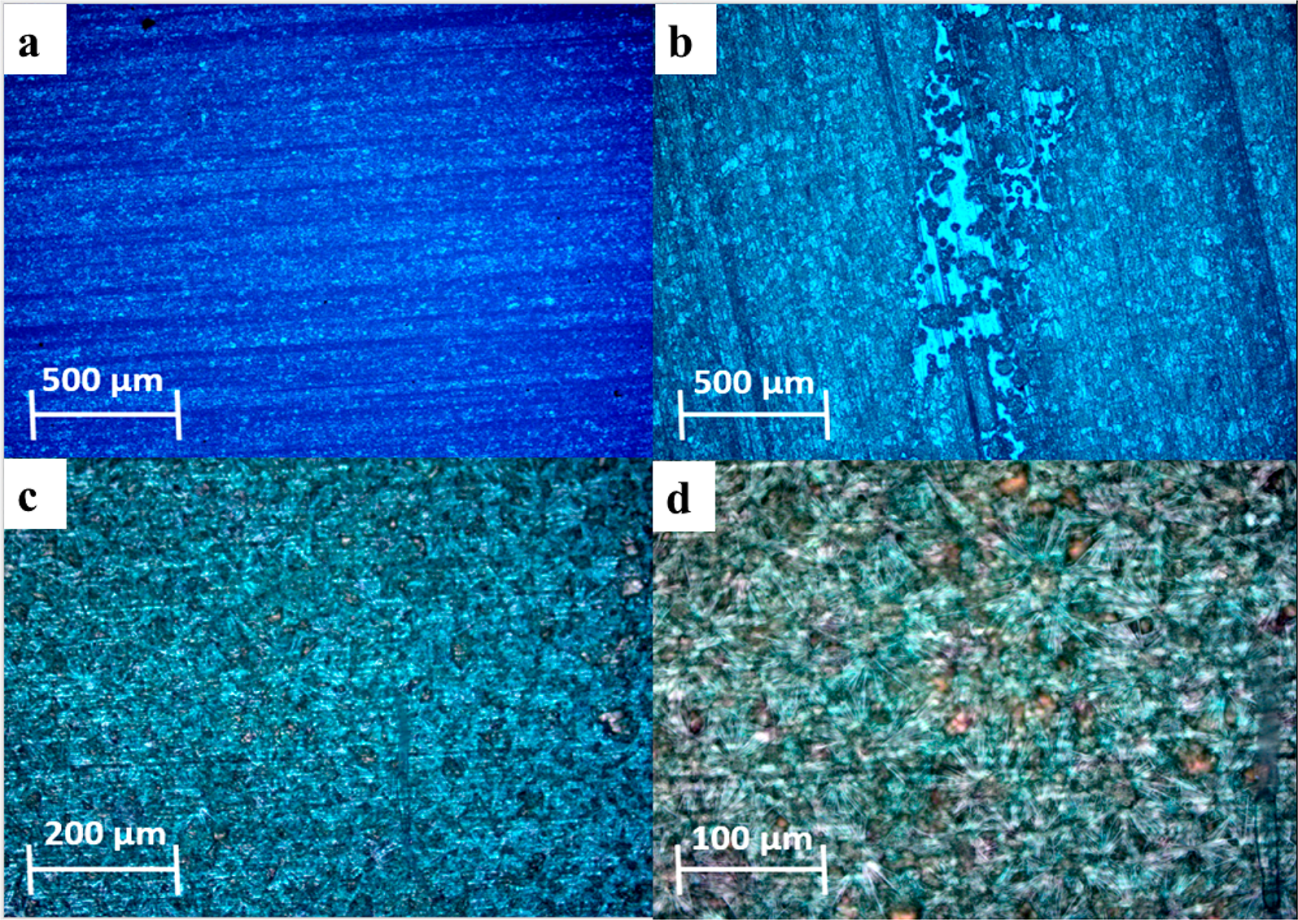
Optical microscopy of polycrystalline films of the glycine–sulfamic acid cocrystal. (a) Large-area optical micrograph of dense films with large grains and (b) optical micrograph contrasting the film with the underlying copper substrate. (c) Higher-magnification micrograph of dense needle-shaped cocrystals and (d) very high-magnification image of the cocrystal needle clusters.

As the SHG measurements confirmed both the noncentrosymmetric nature of the crystals and the presence of a significant electronic contribution to *d*_33_, a new growth approach was developed to allow a precise measurement of the piezoelectric properties of the cocrystal using PFM and piezometer measurements. Figure 4 shows polycrystalline films of the glycine–sulfamic acid cocrystal grown on thick copper substrates. The cocrystals self-assemble into dense films, either with large flat grains (Figure 4a,b) or as needles that grow out from nuclei (Figure 4c,d).

**Figure 5:**
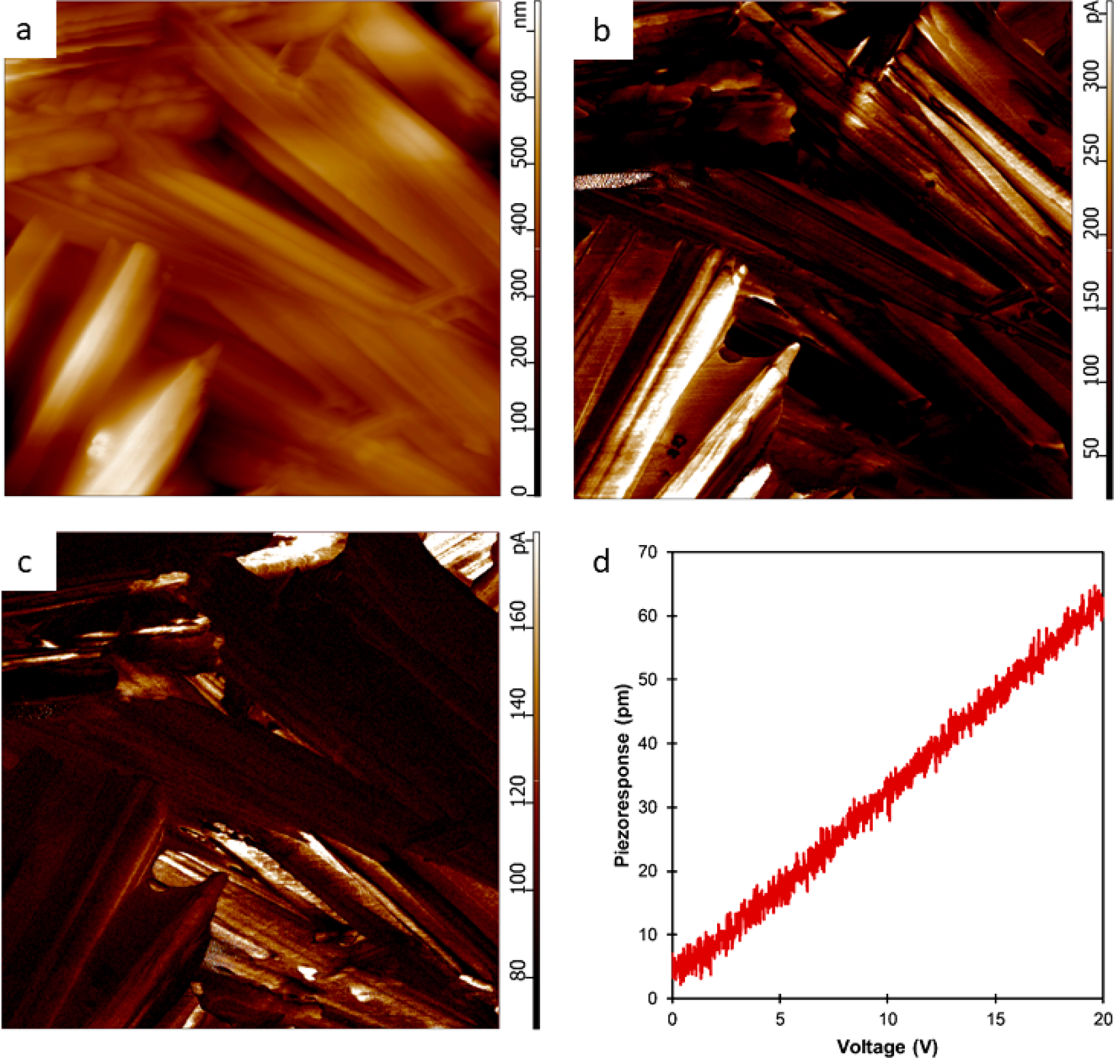
Piezoresponse force microscopy measurements. (a) Topographic image showing criss-crossing arrays of crystals. The height variations are around 700 nm, and the edge length of the image is 10 μm. (b) Corresponding vertical piezoresponse image showing areas of contrast across these crystals. The contrast in the majority of the image is between 200 and 300 pA, which corresponds to a large-area *d*_33_^eff^ value of 2–3 pm/V at 20 V applied AC bias. (c) Lateral piezoresponse image showing areas of contrast complementary to the vertical image. (d) Representative PFM point curve showing the linear relationship between vertical piezoresponse and applied voltage in the cocrystal.

Piezoresponse force microscopy (PFM) measurements on the polycrystalline films shown in Figure 4c,d gave a linear converse piezoelectric effect of 3 pC/N (Figure 5d; slope = 3.00 pm/V; *R*^2^ = 0.99), which gives an estimate of the maximum effective longitudinal piezoelectric coefficient. To further characterize the nanoscale single-crystal response of the cocrystal, we took five independent point measurements across the 700 nm × 700 nm area. These measurements yielded an average *d*_33_^eff^ value of 1.6 ± 0.72 pm/V. The data were acquired using a stiff Pt-coated probe with a spring constant of 19.57 N/m. The gain and input are both equal to 10, and the IOS was determined to be 0.051 at the beginning of the experiment, where IOS is the inverse optical sensitivity, a conversion factor relating the recorded deformation to the desired unit of quantification.^45^

**Figure 6:**
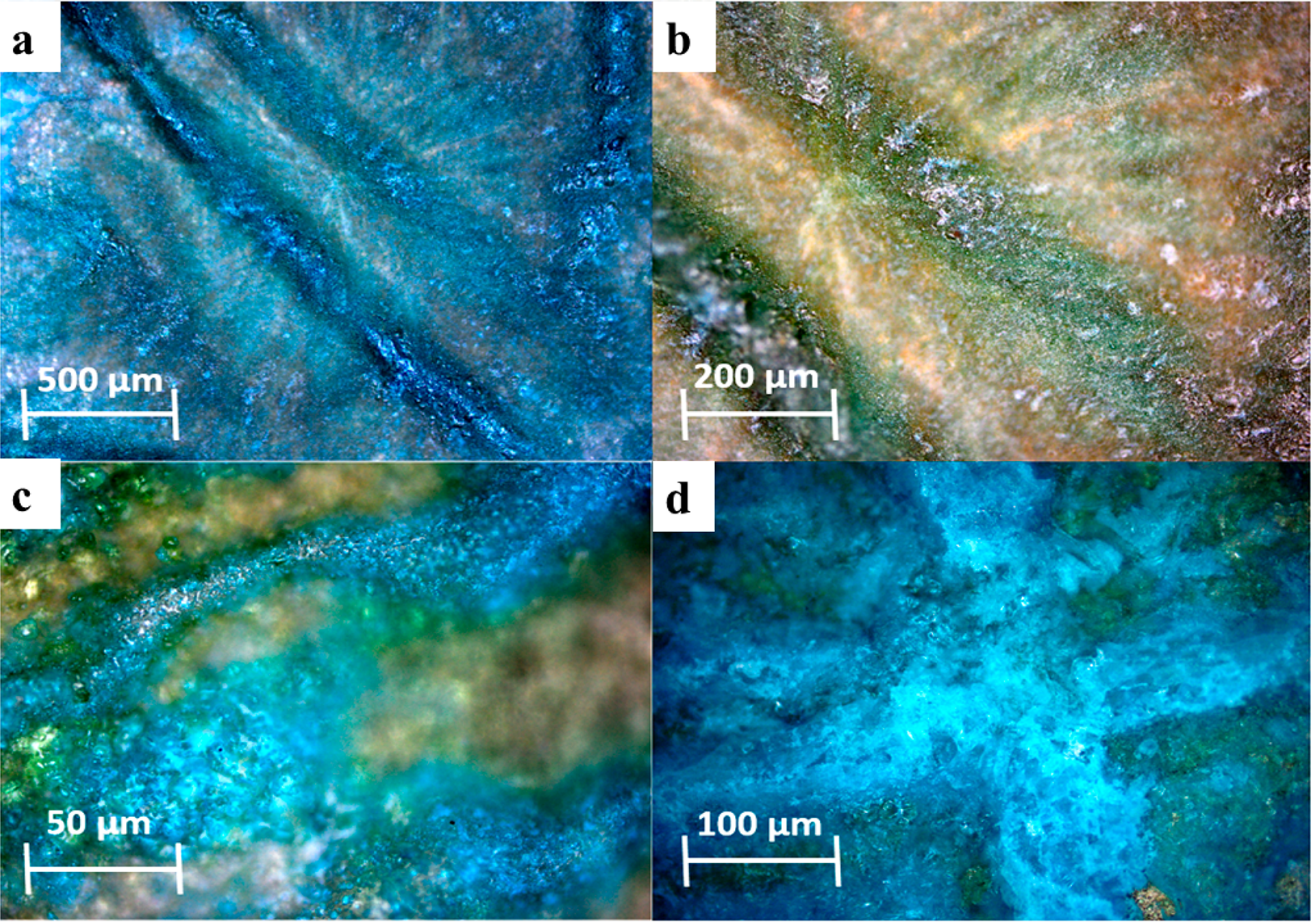
Polycrystalline films of the sulfamic acid coformer. (a) Optical micrograph of sulfamic acid fibrils within a polycrystalline matrix. (b) Higher-resolution image, showing the crystalline nature across all areas of the film. (c) Enlarged micrograph showing the height variation within the film, resulting in distinct crystal topography. (d) A sulfamic acid fibril accidentally damaged during the experiments, revealing a highly crystalline interior.

Finally, we note that the polycrystalline films of the glycine/sulfamic acid cocrystal exhibited a damped but nonzero macroscopic piezoelectric response of ±0.2 pC/N, measured using a commercial piezometer (see Materials and Methods). This order of magnitude lowering of piezoelectric response in polycrystalline films in comparison with single crystals is consistent with our previous work showing a similarly reduced response, due to the effect of compensating dipoles in films of l-threonine, l-alanine, and hydroxy-l-proline.^3^ More recently γ-glycine in polycrystalline form has also demonstrated a 1 order of magnitude dampening of its piezoelectric response.^53^ As a further control, these quasi-static experiments confirm the centrosymmetric nature of the coformer sulfamic acid films, as they exhibited zero piezoelectric response. The sulfamic acid films self-assemble into unusual crystalline tendril structures (Figure 6).

## Conclusions

We demonstrate here that cocrystallization of glycine and sulfamic acid can be used to create noncentrosymmetric cocrystals, using a combination of crystal growth, predictive materials modeling, and nonlinear optical and force microscopy characterization. All methods characterize a significant lateral piezoelectric response in the glycine–sulfamic acid ionic cocrystal and converge to a consensus value of ∼2 pC/N. This cocrystal engineering approach herein endows the nonpiezoelectric α-glycine with new functional properties that were absent in both the pure amino acid and the sulfamic acid. By coupling DFT models with the growth methods and gentle characterization techniques required for soft organic crystals, we could map the influence of the coformer at the nanoscale, advancing our knowledge for future crystal engineering studies. The demonstrated polycrystalline film growth will expand the range of applications for electrically active biomolecular crystals in applications from tissue regeneration to drug delivery and to AI-driven health monitoring.^60^

## Supporting Information Available

XRD data and DSC and TGA graphs (PDF)

## Accession Codes

CCDC 2090689 contains the supplementary crystallographic data for this paper. These data can be obtained free of charge via www.ccdc.cam.ac.uk/data_request/cif, or by emailing data_request@ccdc.cam.ac.uk, or by contacting The Cambridge Crystallographic Data Centre, 12 Union Road, Cambridge CB2 1EZ, UK; fax: +44 1223 336033.
