# Supplementary material for: A Piezoelectric Ionic Cocrystal of Glycine and Sulfamic Acid
Source: Cryst Growth Des. 2021 Sep 27;21(10):5818–27. doi: 10.1021/acs.cgd.1c00702 (PMC8498985; doi:10.1021/acs.cgd.1c00702)
Supplement: Supplementary file 1 — cg1c00702_si_001.pdf [file cg1c00702_si_001.pdf]

Supplementary Information

A piezoelectric ionic cocrystal of glycine and sulphamic acid

Sarah Guerin[1,2], Sanaz Khorasani[1,3], Matthew Gleeson[2], Joseph O'Donnell[2], Rana Sanii[1,3] Reabetswe Zwane[1,4], Anthony M. Reilly[1,4], Christophe Silien[2], Syed A.M. Tofail[2], Ning Liu[2], Michael Zaworotko[1,3], Damien Thompson[1,2*]*

[1]SSPC, Science Foundation Ireland Research Centre for Pharmaceuticals, University of Limerick, V94 T9PX, Ireland

[2]Department of Physics, Bernal Institute, University of Limerick, V94 T9PX, Ireland

[3]Department of Chemical Sciences, Bernal Institute, University of Limerick, V94 T9PX, Ireland

[4]School of Chemical Sciences, Dublin City University, Glasnevin, D09 C7F8 Dublin, Ireland

Supplementary Table 1: Data collection and refinement statistics

**Crystal data**	
Chemical formula	$C_2H_6NO_2$, $C_2H_5NO_2$, H_2NO_3S
Mr	247.23
Crystal system	monoclinic
Space group	P 1 n 1
a (Å)	4.8995
b (Å)	12.190
c (Å)	16.800
α (°)	90
□ (°)	90
γ (°)	90
V (Å$_3$)	1003.4
Z , *Z*'	4
μ(mm^{-1})	0.348
Temperature (K)	173
**Data collection**	
Diffractometer	Bruker APEX-II CCD
Wavelength (Å)	0.71073
$N_{measured}$ (unique)	5002 (3367)
$N_{observed}$ [$I > 2\sigma(I)$]	3176
R_{int}	0.0451
θ_{max} (°)	28.23
**Refinement**	
R [F2 > 2 σ (F2)]	0.0444
wR	0.0508
wR [*F*2 > 2 σ (*F*2)]	0.1057
wR (*F*2)	0.0968
Goodness-of-fit	1.12
No. of reflections	3367
No. of parameters	283
No. of restraints	2

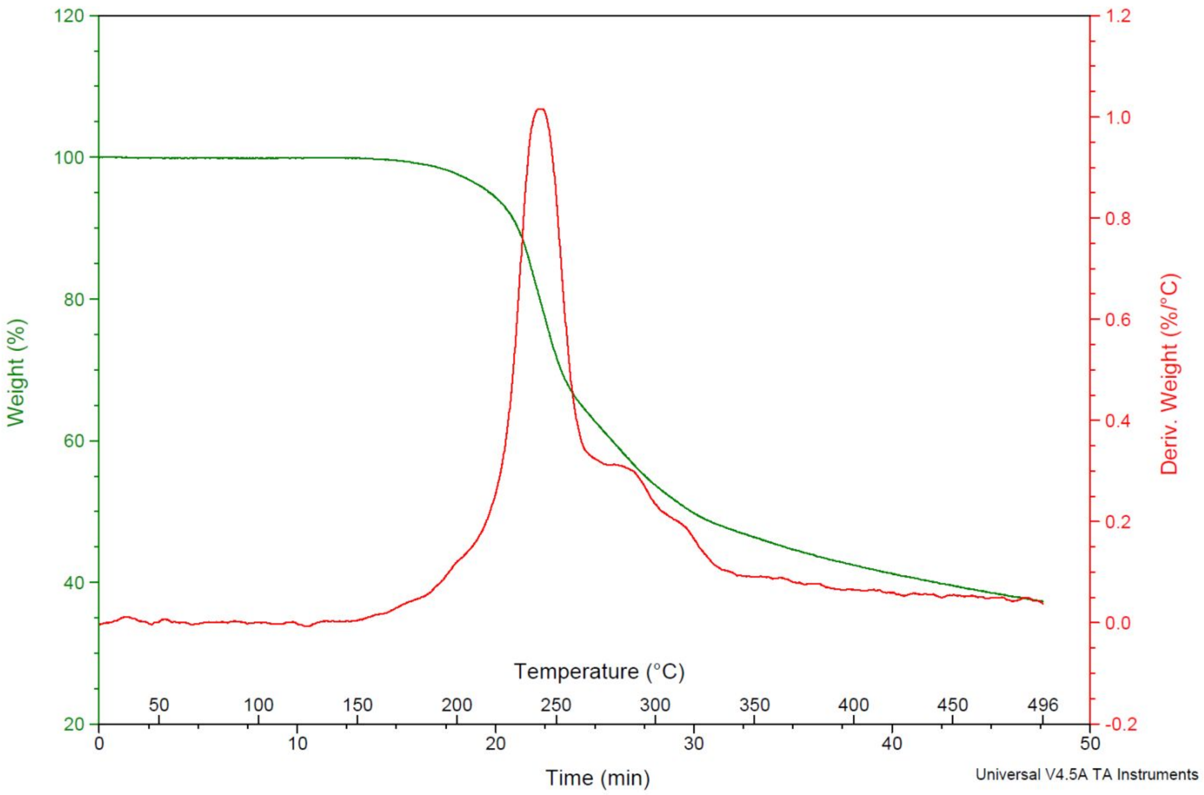

Figure S1: Thermogravimetric Analysis (TGA) of a glycine-sulfamic acid cocrystal.

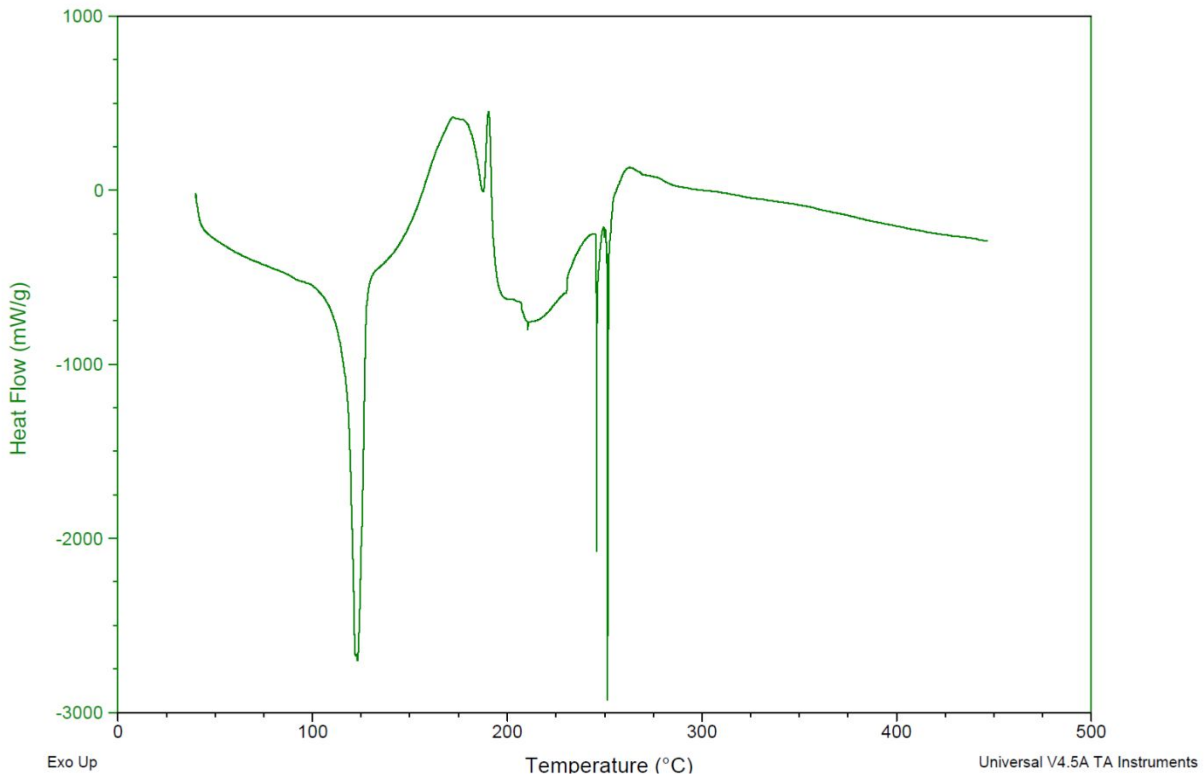

Figure S2: Differential Scanning Calorimetry (DSC) of a glycine-sulfamic acid cocrystal.
